# XooNet: a high-throughput UAV-based approach for field screening of bacterial blight-resistant germplasm in wild rice

**DOI:** 10.3389/fpls.2026.1765317

**Published:** 2026-02-20

**Authors:** Pan Pan, Wenlong Guo, Mingxia Li, Haochun Li, Jingxi Yang, Zhihao Guo, Huibo Zhao, Guoping Yu, Maomao Li, Long Yi, Xiaoming Zheng, Guomin Zhou, Jianhua Zhang

**Affiliations:** 1 National Nanfan Research Institute, Chinese Academy of Agricultural Sciences, Sanya, China; 2 Agricultural Information Institute, Chinese Academy of Agricultural Sciences, Beijing, China; 3 National Agriculture Science Data Center, Beijing, China; 4 College of Agronomy and Biotechnology, China Agricultural University, Beijing, China; 5 Yazhouwan National Laboratory, Sanya, China; 6 China National Rice Research Institute, Hangzhou, China; 7 Rice Research Institute, Jiangxi Academy of Agricultural Sciences, Nanchang, China; 8 Jiangxi Provincial Crop Germplasm Resources Research Center, Nanchang, China; 9 Institute of Crop Science, Chinese Academy of Agricultural Sciences, Beijing, China; 10 Nanjing Institute of Agricultural Mechanization, Ministry of Agriculture and Rural Affairs, Nanjing, China; 11 Institute of Western Agriculture, Chinese Academy of Agricultural Sciences, Changji, Xinjiang, China

**Keywords:** bacterial blight, deep learning, disease-resistant breeding, germplasm screening, UAV, wild rice

## Abstract

Bacterial blight (BB) poses a significant threat to rice production, necessitating efficient screening of resistant wild rice germplasm to facilitate breeding. Traditional methods are labor-intensive and subjective, while existing UAV-based approaches suffer from high costs or incomplete solutions. This study introduces XooNet, a novel UAV-based method for automated BB resistance screening in wild rice, which classifies wild rice into several levels based on BB resistance. To facilitate this method, a high-precision and lightweight oriented bounding box (OBB) detection algorithm for BB in wild rice has been developed. Experimental results show that the screening method achieved an accuracy of 97.5%. After applying the LAMP pruning strategy to balance performance and efficiency, the detection model achieved an accuracy of 93.1% with a significantly reduced parameter size of 1.4M and a computational complexity of 3.5 GFLOPs. This approach will facilitate the high-throughput screening of extensive wild rice germplasm for BB resistance, thereby expediting the discovery of valuable wild rice genetic resources.

## 1 Introduction

Rice (Oryza sativa) is one of the most important staple crops globally, feeding nearly half of the world’s population (Londo et al., 2006; Muthayya et al., 2014; Fukagawa and Ziska, 2019). The spread of bacterial blight (BB) severely impacts both rice yield and quality, with potential reductions exceeding 70% in severe cases (Gnanamanickam et al., 1999; Kumar et al., 2023; Sabar et al., 2024). Currently, fungicides such as thiazole zinc, tebuconazole, and prochloraz are primarily used to control this disease (Shcherbakova, 2019; Gupta and Gupta, 2025). However, these chemical treatments pose risks of environmental contamination and chemical residues (Thompson and Darwish, 2019). The most cost-effective and efficient approach to controlling BB is to identify and utilize resistance genes for breeding disease-resistant rice varieties (Sahu et al., 2022; Jain et al., 2025). Selecting materials with BB resistance is essential for identifying disease resistance genes and breeding disease-resistant rice varieties (Zhang et al., 2020).

Wild rice, through prolonged natural selection, has accumulated numerous resistance genes that enable adaptation to harsh environments, making it a valuable source for identifying BB resistance genes (Ziyi et al., 2022; Zheng et al., 2024; Pan et al., 2025). Key disease-resistant genes such as Xa21 (Song et al., 1995), Xa23 (Wang et al., 2015), and Xa27 (Xu et al., 2024) have been discovered in wild rice and successfully incorporated into rice breeding, significantly enhancing resistance to BB (Amante-Bordeos et al., 1992).

Currently, screening of BB-Resistant Germplasm in Wild Rice is typically conducted by breeding researchers who monitor and record disease progression daily in wild rice disease fields. This method is inefficient, subjective, lacks data consistency, and is not suitable for large-scale field applications (Tannidi et al., 2025). Furthermore, the continuous evolution of Xoo physiologic races and changes in geographic, ecological, and cultivation conditions can lead to a decline in the resistance of highly resistant varieties, requiring ongoing screening efforts (Li et al., 2025; Zhang and Li, 2025). This demands significant time, effort, and resources from researchers (Oliveira-Garcia et al., 2024; Wang et al., 2025). Therefore, there is a pressing need for the development of automated, high-throughput methods for screening BB resistance in wild rice (Pan et al., 2023b).

In the past five years, the rapid development of drones and deep learning has led to studies utilizing UAVs for screening rice disease resistance. (Pan et al., 2023a) developed the Xoo-YOLO detection model for detecting BB in wild rice. Although the model achieved high detection accuracy, it only focused on disease target detection and proposed future applications for screening resistant materials, but this has not yet been realized. Additionally, the model still has room for optimization in terms of size and computational complexity. Another study by (Bai et al., 2023) analyzed spectral data to develop a UAV-based remote sensing model for screening rice germplasm with BB resistance. This model effectively meets the needs of large-scale screening and achieves high accuracy. However, due to the use of hyperspectral equipment, which is costly (over $80,000) and requires operators with high technical expertise, it has not been widely adopted by breeding laboratories.

Although the previous studies have certain limitations, such as high equipment costs, operational complexity, and the lack of an end-to-end screening solution, they have demonstrated the feasibility of using UAVs for screening BB-resistant wild rice materials. This approach has proven effective for large-scale screening with high accuracy (Shaodan et al., 2023; Lu et al., 2025; Zhu et al., 2025). To address the deficiencies in existing studies that limit their large-scale application in breeding, this paper proposes a novel UAV-based screening method for wild rice BB resistance, aiming to accelerate the utilization of wild rice in rice breeding for BB resistance.

The main contributions of this paper are as follows:

(1) A UAV-based screening method for wild rice BB resistance, XooNet, is proposed.
(2) A high-precision and lightweight OBB detection algorithm for BB in wild rice is developed, suitable for this screening method.
(3) The proposed detection model and screening method are evaluated for performance to verify their effectiveness and reliability.

## 2 Materials and methods

### 2.1 Material preparation and images acquisition

The wild rice germplasm was provided by the Yazhou Bay National Laboratory and the Institute of Crop Science, Chinese Academy of Agricultural Sciences, and is stored at the National Wild Rice Germplasm Repository in Sanya, Hainan Province. Samples were collected from regions including Guangxi, Hainan, and Yunnan, encompassing both ordinary and medicinal wild rice varieties. Disease plots were established on February 18, 2023, and March 3, 2024, at the Potianyang base in Yazhou District, Sanya City (Latitude 18°23′36″, Longitude 109°9′52″). Based on their growth status, tillering, and population distribution, 120 and 50 representative and diverse wild rice samples were transplanted. After transplanting, compound fertilizer was applied one week later, with regular water changes, field cleaning, and pest control carried out.

After tillering reached the reproductive stage, wild rice was inoculated with the bacterial blight pathogen Xanthomonas oryzae pv. oryzae (strain PXO99, a highly virulent, widely pathogenic Philippine race 6) following the Rice Bacterial Blight Resistance Identification Technical Standards. The bacteria, strain PXO99, known for its high virulence and broad pathogenicity, were cultured on PSA medium (10 g/L tryptone, 10 g/L sucrose, 1 g/L monosodium glutamate, 15 g/L agar, pH 7.0) for 2 days, then washed with sterile water and diluted to an OD600 of 0.6. Using scissors dipped in the bacterial solution, leaves of individual wild rice plants were cut 3–5 cm from the leaf tip, with at least 7–10 leaves inoculated per sample. Jingang 30, a susceptible variety, was used as a positive control, with inoculation considered valid only if the control exhibited symptoms.

RGB images of rice infected with BB were captured at different time points using DJI Mini 2 (DJI Innovations, Shenzhen, China) and DJI Mini 4 Pro (DJI Innovations, Shenzhen, China) UAVs. The flight altitude was set between 0.6 m and 1.5 m above the wild rice field, with the camera gimbal pitch adjusted to -90° to -60°. The images had a resolution of 1920×1080 pixels and were saved in JPG format. Image acquisition occurred between 5 and 21 days post-infection, during the morning (8:00–11:00) and afternoon (16:30–18:30), under weather conditions such as sunny, cloudy, and overcast, with ambient temperatures ranging from 22 °C to 30 °C. All images were taken under natural field conditions with ambient lighting, without the use of a flash. The images may contain varying degrees of obstruction, water surface reflection, overexposure, as well as noise from weeds, dead leaves, bird droppings, and other field debris. Each image contained between 1/4 and 2 wild rice plants. In total, 2,035 images were captured. Their quality and accuracy for representing BB symptoms were verified by two wild rice germplasm identification experts, confirming that the depicted lesions were definitively BB.

Data processing of the UAV images was carried out to ensure high-quality input for the model. To mitigate noise, overexposure, and occlusion, specific preprocessing techniques were applied. A Gaussian blur with a kernel size of 5×5 and a standard deviation (σ) of 1.5 was applied to reduce high-frequency noise. Morphological opening operations using a 3×3 rectangular structuring element were employed to clean small debris and refine the boundaries of detected lesions. Additionally, Z-score normalization was performed to standardize lighting conditions across the images by subtracting the mean and dividing by the standard deviation of the dataset, thereby reducing the effect of varying weather and light conditions during UAV capture.

**Figure 1:**
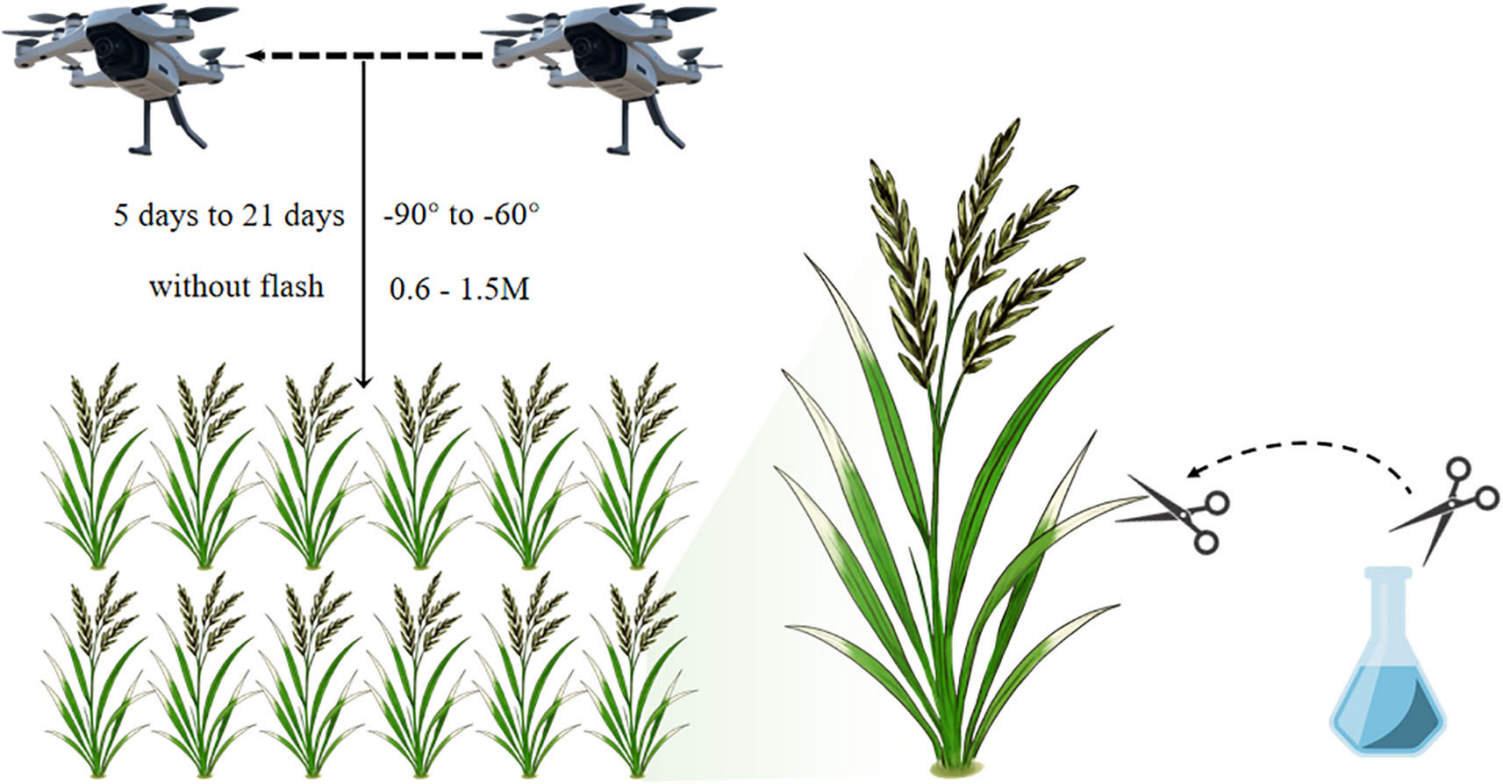
The process of material preparation and image acquisition.

**Figure 1** illustrates the process of material preparation and image acquisition.

### 2.2 Image preprocessing and dataset construction

To meet the input and training requirements of deep learning models, this study employed an image cropping strategy, dividing the raw UAV-captured images into multiple 640×640 pixel blocks. A sliding window method with a fixed step size was used to ensure that each cropped region covered different parts of the original image, creating independent training samples. Additionally, to enhance dataset diversity and model robustness, data augmentation techniques were applied, including random rotations, horizontal and vertical flips, scaling, and color adjustments. This resulted in a total of 12,210 images. The dataset, comprising images collected from both the 2023 and 2024 growing seasons, was pooled together and randomly shuffled. It was then split into training (9,768 images), validation (1,221 images), and testing (1,221 images) sets in an 8:1:1 ratio. This random splitting strategy ensures that the model learns from a diverse range of environmental conditions present in both years. The BB lesion areas on wild rice were manually annotated using the open-source software roLabelImg, with the annotation information saved in.xml format.

### 2.3 BB detection model

#### 2.3.1 Overall model

YOLOv11, introduced in 2024, is an advanced object detection model that offers various versions, including the standard YOLOv11, YOLOv11-OBB, YOLOv11-Seg for segmentation tasks, and YOLOv11-Pose for pose estimation (Jegham et al., 2024; Pan et al., 2024b; Hidayatullah et al., 2025). Additionally, it comes in multiple size variants: n, s, l, and x, with each variant optimized for different levels of computational efficiency and performance (Khanam and Hussain, 2024).

**Figure 2:**
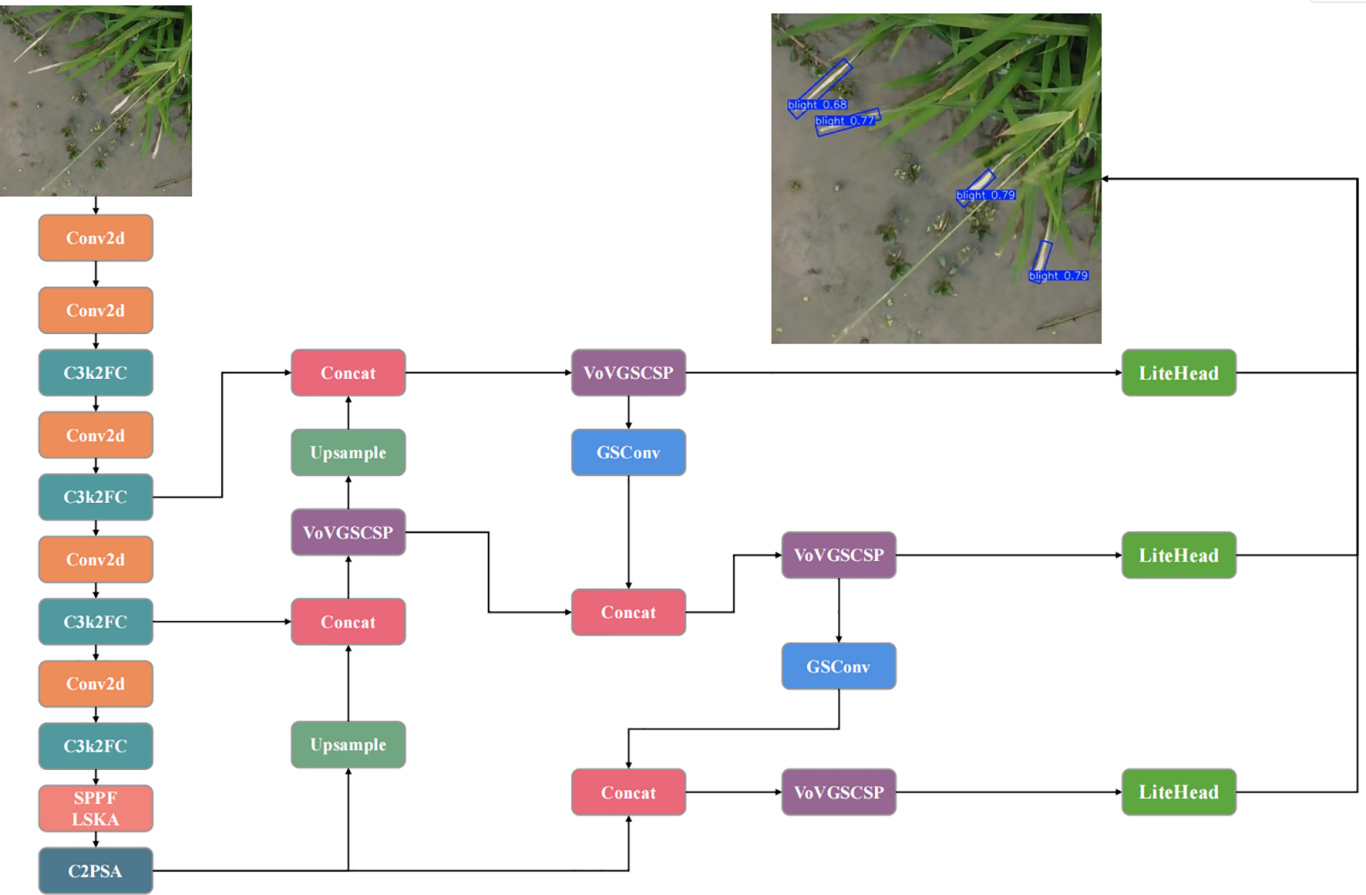
Structure of the BB detection model.

The UAV captures images from above, often presenting targets at various angles. Additionally, bacterial blight (BB) typically manifests as elongated lesions. Considering these factors, the YOLOv11-OBB model was selected. To optimize model efficiency and reduce computational complexity, the smallest variant, the n model, was chosen as the foundation for improvements. As shown in **Figure 2**, the improved model’s overall structure consists of three main components: Backbone, Neck, and Head.

The key improvements are outlined below:

1. C3k2FC Module in Backbone: Integrates PConv and CGLU into the C3k2 structure to reduce redundancy and enhance efficiency.
2. SPPF_LSKA Module in Backbone: Incorporates LSKA post-pooling to improve multi-scale feature aggregation and reduce noise.
3. SlimNeck in Neck: Utilizes GSConv and VoVGSCSP to optimize feature fusion while minimizing FLOPs.
4. LiteHead in Detection Head: Employs separated Batch Normalization (BN) and dynamic anchors for improved handling of rotations.
5. LAMP Pruning: Implements adaptive global pruning to compress model parameters while maintaining accuracy.

#### 2.3.2 C3k2FC

The C3k2 module in YOLOv11-OBB, based on the Cross-Stage Partial (CSP) structure, splits the input feature map into two parallel branches. This design enhances feature reuse and gradient flow by repeating bottleneck units, supporting flexible configurations of layers and channels to balance model depth and computational efficiency. However, this module suffers from redundant convolution operations, increasing computational cost and complicating deployment on resource-constrained UAV embedded systems.

To address these issues, this paper integrates the FasterNet Block’s Partial Convolution (PConv) and the TransNext CGLU module to enhance the C3k2 module in both the Backbone and Neck networks (Chen et al., 2023; Pan et al., 2024a; Shi, 2024). This modification aims to achieve a lightweight design, reduce computational redundancy, and minimize parameter size, making it more suitable for UAV embedded systems with limited resources. The CGLU module, an evolution of the GLU gated linear unit, extends local spatial perception by inserting a 3×3 depthwise convolution before the activation function in the gating branch. The module consists of two linear transformation paths: one path applies a 3×3 depthwise convolution followed by a gated activation function to strengthen local feature capture, while the other generates the baseline output. These paths are merged via element-wise multiplication, adjusting feature weights, and the final output is produced through linear projection and a residual connection with the input. This design optimizes the gating signal using local information from the depthwise convolutions, adjusting inter-channel dependencies. The FasterNet Block simplifies the network using an inverted residual structure, starting with a 1×1 pointwise convolution for channel expansion and feature compression. It then inserts the PConv module, which applies a 3×3 standard convolution to only a quarter of the input channels for spatial feature mixing, while the remaining channels are passed through identity mapping to reduce full-channel computation. Following this, a normalization layer and ReLU activation function maintain feature distribution and non-linearity, concluding with another 1×1 pointwise convolution for channel restoration and output integration.

**Figure 3:**
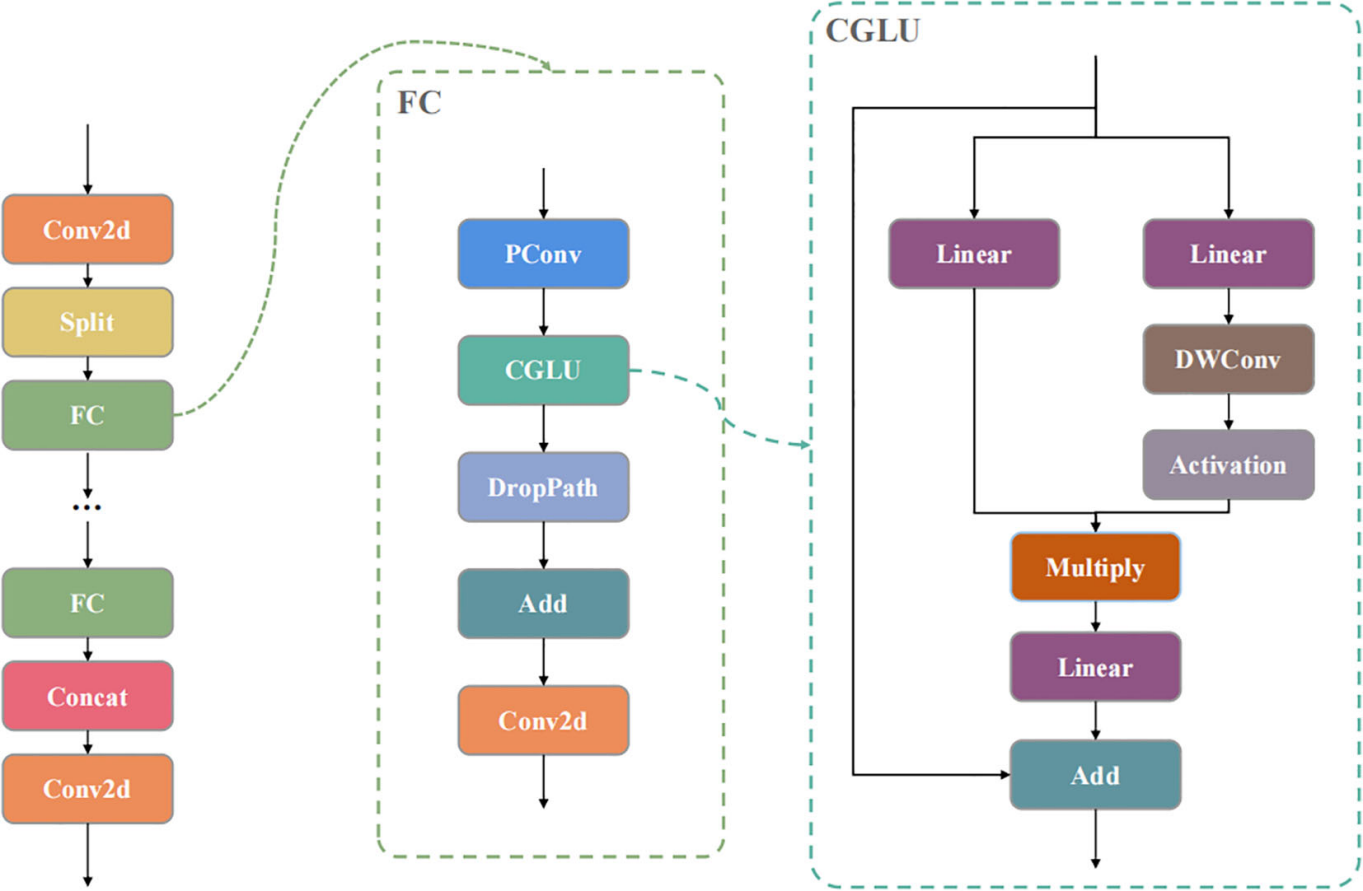
Structure of C3k2FC.

In this study, we replace the two 1×1 pointwise convolutions in the FasterNet Block with the CGLU module, forming an improved FC structure. We also integrate PConv into the depthwise convolution section of the C3k2 bottleneck, resulting in the improved C3k2FC module, as shown in **Figure 3**. This structure optimizes feature extraction through partial channel convolutions and gating mechanisms, reducing floating-point operations and memory access. The FC Block, with the PConv module, extracts multi-scale features from the input image while maintaining low computational overhead. Combined with the CGLU module, the design supports dynamic feature weight allocation. The improved C3k2FC module integrates these components to capture both detailed and global information, supporting wild rice BB detection and localization from UAV perspectives, and is suitable for edge device deployment.

#### 2.3.3 SPPF_LSKA

The SPPF module in YOLOv11, based on the spatial pyramid pooling (SPPF) structure, aggregates multi-scale features by applying maximum pooling operations of different sizes in parallel. These features are then fused through channel concatenation and convolution to extract global context. The module aims to reduce information loss and enhance adaptability to targets of varying scales, supporting efficient feature representation. However, the pooling operations in this module have limitations, leading to inefficient feature aggregation in complex scenes and the potential introduction of background noise. This is especially problematic when dealing with UAV-captured rice field images that involve varying lighting, leaf occlusion, and resolution fluctuations, which may cause fine-grained details to be overlooked or irrelevant features to be amplified, resulting in false detections and degraded performance.

To address these challenges, we propose the integration of the Large Separable Kernel Attention (LSKA) mechanism to improve the SPPF module in the Backbone network, achieving a lightweight design while enhancing multi-scale feature extraction and local attention capabilities. The LSKA module, based on separable convolutions, decomposes large kernel convolutions into 1D separable operations in the horizontal and vertical directions to simulate large receptive field attention. This approach, combined with depth-wise separable convolutions, reduces parameter count.

**Figure 4:**
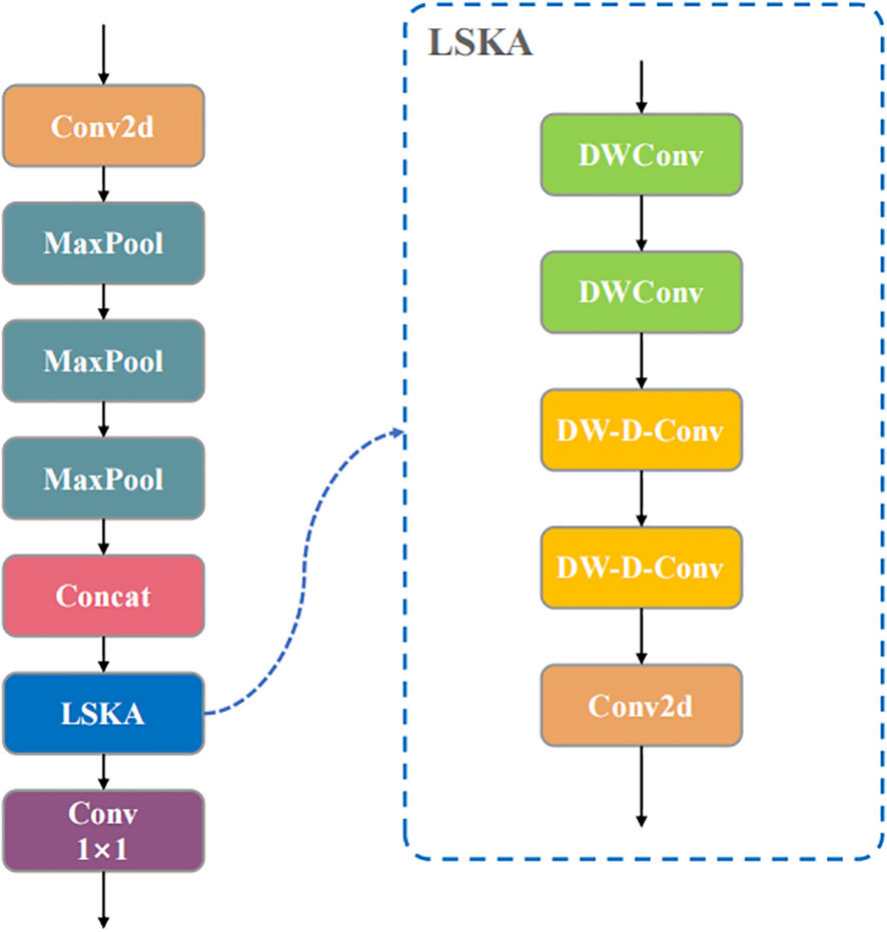
Structure of SPPF_LSKA.

The SPPF_LSKA module integrates the LSKA mechanism (Lau et al., 2024) after the pooling layers of the traditional SPPF, as shown in **Figure 4**. The structure first extracts multi-resolution features through multiple maximum pooling branches, then applies the LSKA module to perform attention weighting on the concatenated features. The horizontal and vertical branches of the LSKA handle spatial information, and the depthwise separable convolution further optimizes channel mixing. Finally, the fused features are output through a 1×1 convolution to enhance both global and local semantic representations.

This study replaces the original SPPF in YOLOv11-OBB with the improved SPPF_LSKA module, optimizing multi-scale feature integration through attention mechanisms to support wild rice BB detection and localization from UAV perspectives.

#### 2.3.4 SlimNeck

The standard convolution (SC) component in YOLOv11-OBB captures features by processing multi-channel data through parallel multi-kernel operations. While this promotes deep interactions between channels, it also leads to parameter inflation and a significant increase in floating-point operations (FLOPs), which limits real-time performance, especially on resource-constrained UAV embedded systems. On the other hand, efficient architectures like MobileNet and ShuffleNet alleviate the computational burden by using depthwise separable convolution (DSC). However, DSC isolates channel data during computation, which significantly reduces feature integration and extraction effectiveness. This limitation is problematic for wild rice BB detection from UAV perspectives, where noise from lighting changes, leaf occlusion, and resolution fluctuations can lead to missed or mislocalized small lesions.

To accelerate computation without compromising detection accuracy, this study integrates the GSConv composite convolution unit into the Neck structure of YOLOv11-OBB. The GSConv unit incorporates a Shuffle mechanism to merge the channel association data derived from SC with the spatial output from DSC (Li et al., 2022). Compared to DSC alone, GSConv reduces computational burden while retaining potential correlations, optimizing both accuracy and response time—ideal for crop disease detection on edge hardware.

The core components of the GSConv unit include Conv, DWConv, Concat, and Shuffle operations. The architecture is constructed through the following process: The input feature matrix with C1​ channels is first processed by applying DSC to half of the channels to capture spatial details, while SC is applied to the other half for channel fusion. The outputs from both sides are then concatenated along the channel axis. Subsequently, a Shuffle operation is applied to the concatenated result to promote random channel reorganization and enhance data interaction. The final output matrix contains C2 channels. The VoVGSCSP, an iterative bottleneck combination for GSConv, splits the input matrix channels into two segments. One segment undergoes Conv preprocessing and is passed through a series of GS bottleneck units to extract features, while the other segment serves as a residual path with only a single Conv transformation. This unit, leveraging the CSP framework, enhances feature reuse, employs a one-shot aggregation method to minimize data loss, and integrates batch normalization and SiLU activation to maintain feature consistency.

Finally, this study reconstructs the Neck framework of YOLOv11-OBB using GSConv and VoVGSCSP to form the SlimNeck architecture. These modifications reduce the model’s computational burden, enabling faster inference and data processing while enhancing multi-resolution feature integration. This optimization strikes an effective balance between speed and accuracy.

#### 2.3.5 LiteHead

The detection head of YOLOv11-OBB uses a shared convolution mechanism to process multi-scale feature outputs for boundary box predictions. While this promotes parameter reuse, the use of unified Batch Normalization (BN) can lead to inaccurate moving averages when there are significant statistical differences between features at different levels. This can negatively affect training stability and generalization performance. Additionally, the static anchor box design struggles to adapt to the rotation and scale variations of BB lesions, under the challenges posed by UAVs, such as leaf occlusion, resolution fluctuations, and changing lighting conditions. This often results in localization errors or missed small targets, limiting overall detection performance.

To address these issues, this paper introduces the LiteHead detection head. This optimization is designed to improve computational efficiency, reduce parameter size and operational load, and make the system more suitable for resource-constrained UAV embedded systems. The LiteHead detection head mitigates BN-related issues by separately processing statistical differences between features at different levels, ensuring independent sliding averages and preventing bias accumulation in shared parameters. Additionally, the dynamic anchor box generator adapts the scale, angle, and aspect ratio of anchors based on input features, enhancing the alignment with rotating targets.

The core components of the LiteHead detection head include: a shared convolution layer for multi-task reuse to reduce redundant parameters; a separated BN module that applies BN independently to each detection branch, optimizing feature distribution and speeding up convergence; and a dynamic anchor box generator that computes anchor parameters in real-time using feature map statistics, supporting the prediction of rotated bounding boxes (OBBs). The workflow is as follows: multi-scale feature maps are processed by the shared convolution to extract boundary box regression and classification scores; separated BN standardizes each layer’s output; and the dynamic anchor box module generates adaptive anchors based on feature clustering or gradient guidance, ultimately outputting the OBB predictions.

In this study, LiteHead is integrated into the detection head of YOLOv11-OBB by replacing the standard shared convolution and static anchor boxes. This design optimizes the feature processing pipeline, reduces floating-point operations and memory access, and enhances the ability to capture rotational details and global information. The LiteHead architecture is well-suited for BB detection and localization in UAV-captured rice field images.

#### 2.3.6 LAMP

Despite the introduction of lightweight modules, the YOLOv11-OBB model still faces parameter redundancy and computational intensity, resulting in increased floating-point operations (GFLOPs) and memory requirements. This issue is particularly problematic for resource-constrained UAV embedded systems, where it can lead to inference delays and deployment bottlenecks.

**Figure 5:**
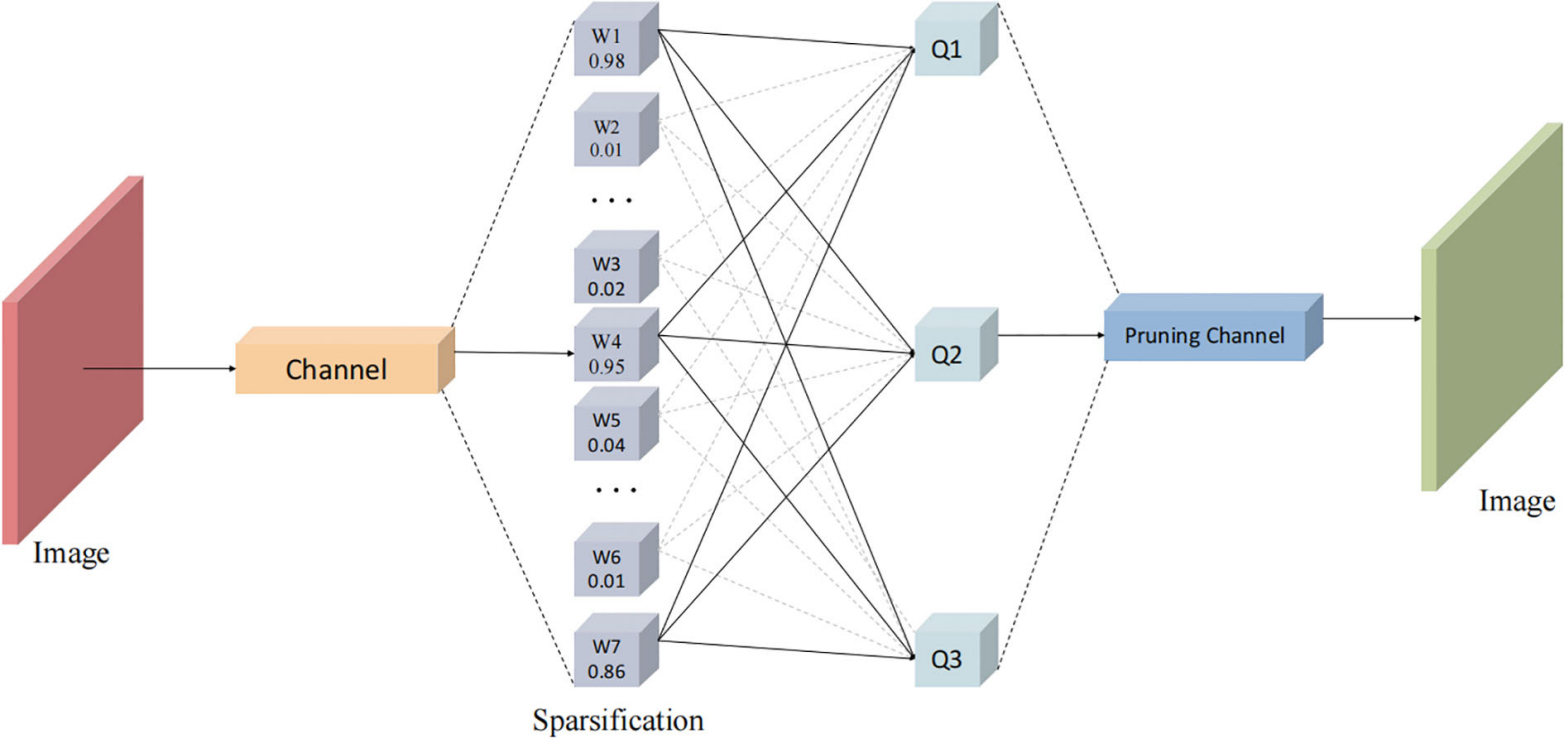
Process of LAMP.

To address these challenges, this study introduces Layer-adaptive Magnitude-based Pruning (LAMP) to further optimize the YOLOv11-OBB model (Lee et al., 2020). LAMP performs global pruning to reduce both parameter size and computational overhead, making the model more suitable for the computational constraints of UAV platforms. LAMP is a magnitude-based pruning strategy that calculates a layer-adaptive importance score (LAMP score) to determine pruning thresholds. This avoids performance degradation due to uniform sparsity and dynamically adjusts the sparsity of each layer based on the statistical characteristics of the weight distribution. As shown in **Figure 5**, early layers retain more connections to capture fine-grained features, while later layers undergo more aggressive pruning to reduce complexity.

The LAMP pruning process proceeds in four stages: pre-training the complete model to obtain initial weights; calculating the global LAMP scores to sort all weights and set thresholds according to the target sparsity; applying magnitude-based pruning layer-by-layer to remove connections below the threshold; and finally fine-tuning the pruned model to restore accuracy, typically using techniques like knowledge distillation or learning rate scheduling.

Compared to traditional magnitude pruning, LAMP improves accuracy retention after pruning by using the layer-adaptive mechanism. This allows for significant reductions in parameters and GFLOPs without sacrificing detection performance.

In this study, LAMP is applied to the Backbone, Neck, and Detection Head of YOLOv11-OBB, resulting in a lightweight variant through iterative pruning and fine-tuning. This optimized structure enhances resource utilization and supports the capture of both fine details and global information, making it well-suited for wild rice BB detection and localization from UAV perspectives.

### 2.4 Screening method

**Figure 6:**
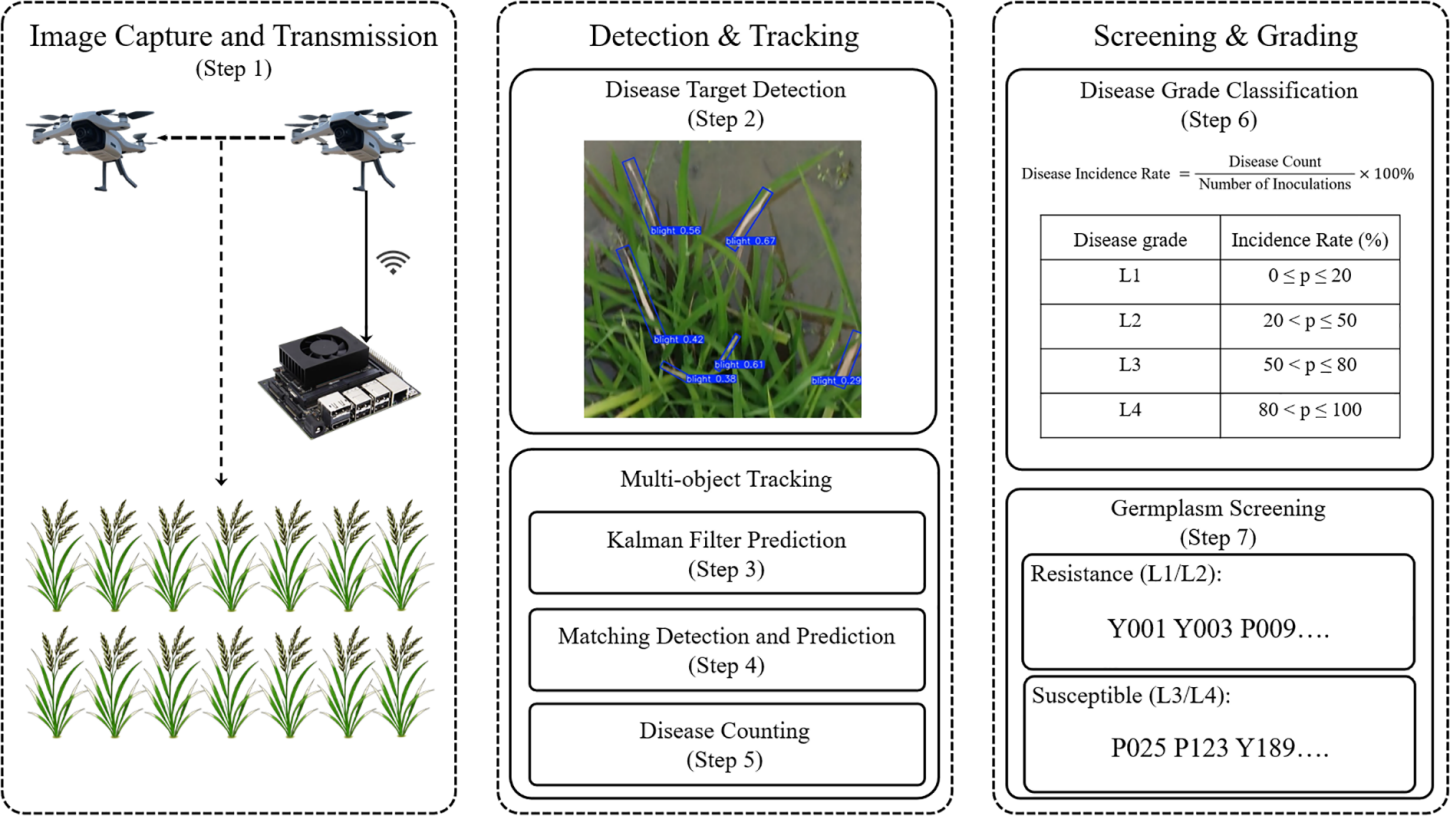
The overall framework of the proposed XooNet method.

The continuous movement of UAVs during video capture often results in partial occlusion of wild rice plants or the redundancy of the same BB target appearing across multiple frames. Consequently, relying solely on static detection from individual images can lead to missed, incorrect, or duplicate counts, thereby compromising the accuracy and stability of the screening process. To address these challenges, we propose an integrated screening workflow. As illustrated in **Figure 6**, this method consists of the following seven steps:

#### 2.4.1 Image capture and transmission

The user controls the UAV to fly over the wild rice disease field, adjusting the camera’s focus and gimbal angle, and then activates the recording function. The captured video data is transmitted to the edge computing device via either wireless or wired connection.

#### 2.4.2 Disease target detection

The video recorded by the UAV is processed through the BB detection model proposed in this paper, which detects and generates corresponding Oriented Bounding Boxes.

#### 2.4.3 Kalman filter prediction

A multi-object tracking model is introduced, utilizing the Kalman Filter to predict the location of targets. The Kalman Filter is a recursive estimation algorithm that uses statistical inference based on historical data to estimate the current position of a target. This prediction provides prior information for disease detection in subsequent frames, reducing bias due to occlusion or detection errors.

The Kalman filter state update equation is expressed in Equation 1:

(1) $$\begin{array}{c} {\text{x}}_{t|t-1}=F\cdot {\text{x}}_{t-1|t-1}+B\cdot {\text{u}}_{t} \end{array}$$

where ${\text{x}}_{\text{t}|t-1}$ represents the predicted state at time t, F is the state transition matrix, B is the control input matrix, and ${\text{u}}_{\text{t}}$ is the control vector. This prediction helps improve the accuracy of the detection in the following frames.

#### 2.4.4 Matching detection and prediction

The Hungarian Algorithm is used to match the current frame’s detection results with the Kalman Filter’s predicted positions. The matching score, representing the similarity between targets, is calculated as shown in Equation 2:

(2) $$\begin{array}{c} score\left(i,j\right)=\lambda \cdot {\text{IoU}}_{OBB}\left(B_{i}^{t-1},B_{j}^{t}\right)+\left(1-\lambda \right)\cdot {\text{Sim}}_{\text{cos}}\left(F_{i}^{t-1},F_{j}^{t}\right) \end{array}$$

where IoU_OBB represents the Intersection over Union calculated based on the Oriented Bounding Boxes (OBB) to accurately account for the rotation of the long, narrow lesions. ${\text{Sim}}_{\text{cos}}$ represents the Cosine Similarity, derived from the Cosine Distance (calculated as 1 - Cosine Distance), between the re-identification (ReID) feature vectors. The feature vectors (*F*) are 128-dimensional embeddings extracted using a lightweight CNN branch integrated into the detection head. λ is a weight parameter balancing spatial overlap and appearance consistency; based on empirical testing on the validation set, λ was set to 0.7.

#### 2.4.5 Disease counting

The BB targets in the tracking list, each with a unique identifier, are counted for BB occurrences.

#### 2.4.6 Disease grade classification

Based on the disease count and the number of disease inoculations in wild rice, the disease incidence rate is calculated using Equation 3:

(3) $$\begin{array}{c} \text{Disease Incidence Rate }=\frac{\text{ Disease Count }}{\text{ Number of Inoculations }}\times 100\% \end{array}$$

The incidence rate is then used to classify wild rice germplasm into BB-resistant categories, as shown in **Table 1**.

**Table 1:** Classification criteria for BB-resistant wild rice germplasm.

Disease grade	Incidence rate (%)
L1	0≤ p ≤ 20
L2	20< p ≤ 50
L3	50< p ≤ 80
L4	80< p ≤ 100

#### 2.4.7 Germplasm screening

Wild rice classified as L1 or L2 is selected as prime candidates for resistance to BB.

### 2.5 Configuration of experimental environment

The experiments were conducted on a Dell tower workstation (Dell, Inc., Round Rock, Texas, USA) running Windows 11. The system was powered by a 12th Gen Intel(R) Core(TM) i5–12500 processor with a clock speed of 3.00 GHz, supported by 64GB of RAM and a 1TB solid-state drive. For GPU-accelerated computations, an NVIDIA GeForce RTX 3080 graphics card (NVIDIA Corporation, Santa Clara, California, USA) with 10GB of video memory was used. The software environment included Python version 3.8.17, along with PyTorch 1.13.0, Torchvision 0.14.0, and CUDA 11.7.

During the model calibration process, hyperparameter optimization was conducted on the validation set. The hyperparameters tested included the learning rate, batch size, and optimizer settings. The Adam optimizer was used with an initial learning rate of 1e-3, a maximum learning rate of 1e-5, a momentum coefficient of 0.937, and a weight decay parameter of 5e-4. The batch size was set to 8, and the input images were resized to a resolution of 640×640 pixels.

For model validation, 5-fold cross-validation was performed to assess the model’s robustness. The dataset was divided into 5 subsets, and the model was trained and evaluated 5 times, each time using a different subset as the validation set and the remaining subsets for training. This process provided a reliable estimation of the model’s performance and ensured that the model generalizes well to unseen data.

The experimental procedure involved 300 epochs for each training run. The training parameters and dataset were kept consistent across all models throughout the training phase to ensure a fair comparison.

In the experiment on performance in edge computing devices, we first converted the model’s weight file to ONNX format, then optimized and added model nodes. Next, we used TensorRT to accelerate the model and deployed it on the Nvidia Jetson Nano. This compact, high-performance AI embedded development board, developed by Nvidia, features a quad-core ARM A57 CPU (1.43 GHz), a 128-core Maxwell GPU, and 4 GB of memory. The operating system used is Ubuntu 18.04, with the environment configured to JetPack 4.6, CUDA 10.2, cuDNN 8.2, and TensorRT 8.0.

### 2.6 Field application test method

To evaluate the performance of the proposed method, video data of 120 wild rice plants inoculated with BB, captured by UAVs in March 2023, were selected. The data were collected at 8, 10, 13, and 15 days post-inoculation. The results from the proposed method were compared with the manual counts performed by breeding experts on-site, providing a systematic assessment of the method’s accuracy.

To validate the effectiveness and stability of the screening method, a field application test was conducted on July 1, 2024, at the Potianyang Experimental Base in Yazhou District, Sanya City, Hainan Province. This test assessed the disease condition of BB in 50 different wild rice samples. The participants included eight individuals: rice disease resistance breeding researchers, experts, local farmers responsible for managing the wild rice base, and graduate students in related fields. During the test, participants were divided into two groups: the validation group, composed of four rice disease resistance breeding researchers and local farmers, performed manual counting to assess the disease condition of the wild rice; the test group, consisting of two graduate students, operated the UAV to collect wild rice images from a height of 0.6 to 1.5 meters and utilized the proposed method for disease condition evaluation. The tests were conducted in the field, with no more than a 30-minute interval between the counting processes of both groups. Two rice disease resistance breeding experts supervised the testing procedures and results.

## 3 Results

### 3.1 Experimental setup and evaluation metrics

To ensure a comprehensive and transparent assessment of the proposed method, the dataset details, grading criteria, and evaluation metrics are consolidated below before presenting the experimental results.

#### 3.1.1 Dataset overview

The dataset used in this study consists of 12,210 images derived from UAV-captured footage of wild rice fields. These images were pooled from data collected in 2023 and 2024 and randomly split into training (9,768 images), validation (1,221 images), and testing (1,221 images) sets in an 8:1:1 ratio.

Grading Criteria: The core objective is to screen wild rice germplasm for bacterial blight resistance. The resistance level is determined based on the disease incidence rate, calculated as the ratio of infected leaves to the total number of inoculated leaves (Eq. 3). The classification standards are detailed in **Table 1**.

#### 3.1.2 Evaluation metrics

The performance of the detection model was evaluated using standard object detection metrics: Precision, Recall, and mean Average Precision (mAP). Additionally, model complexity was assessed using the number of Parameters (Params) and floating-point operations (GFLOPs).

Precision measures the ratio of true positive predictions out of all instances predicted as positive by the model. Recall, on the other hand, quantifies the proportion of true positive samples that the model correctly detects, compared to the total number of actual positive instances. The respective formulas are presented in Equations 4, 5:

(4) $$\begin{array}{c} Precision=\frac{\text{TP}}{\text{TP}+\text{FP}} \end{array}$$

(5) $$\begin{array}{c} Recall=\frac{\text{TP}}{\text{TP}+\text{FN}} \end{array}$$

where True Positive (TP) refers to correctly predicted positive instances, False Positive (FP) refers to negative instances incorrectly predicted as positive, and False Negative (FN) refers to positive instances misclassified as negative.

Average Precision (AP) represents the area under the Precision-Recall curve, summarizing the model’s ability to balance precision and recall at various decision thresholds. Mean Average Precision (mAP) is the average of the AP values across all object categories, providing an overall evaluation of the model’s detection accuracy. These are calculated as shown in Equations 6, 7:

(6) $$\begin{array}{c} AP=\int_{0}^{1}p\left(r\right)dr \end{array}$$

(7) $$\begin{array}{c} mAP=\frac{\sum_{i=1}^{N}APi}{N} \end{array}$$

where n represents the total number of object classes. In this study, n=1.

Params refers to the total number of trainable parameters in the model, indicating its complexity. GFLOPs, or Giga Floating-point Operations, measures the computational cost required to perform one forward pass through the network. A model with fewer Params and lower GFLOPs is more efficient.

### 3.2 Evaluation of the BB detection model’s performance

#### 3.2.1 Ablation experiment

To validate the effectiveness of each proposed module in the improved YOLOv11-OBB model for BB detection in UAV-captured rice field images, we conducted ablation experiments. The baseline is the original YOLOv11-OBB (n variant), and we progressively integrated the proposed modules: C3k2FC, SPPF_LSKA, SlimNeck, and LiteHead. The results are summarized in **Table 2**.

**Table 2:** Ablation study results.

BaseLine	C3k2FC	SPPF_LSKA	SlimNeck	LiteHead	mAP	FLOPS/G	Params/M
✓					88.4%	6.7	2.6
✓	✓				90.1%	6.1	2.3
✓	✓	✓			92.6%	6.4	2.6
✓	✓	✓	✓		93.1%	6.2	2.6
✓		✓			93.9%	6.9	2.9
✓		✓	✓		94.4%	6.5	2.9
✓		✓	✓	✓	93.6%	6.7	3.1
✓			✓		91.7%	6.3	2.6
✓			✓	✓	91.9%	6.0	2.4
✓				✓	92.8%	6.4	2.4
✓	✓	✓	✓	✓	94.1%	5.9	2.5

The integration of C3k2FC demonstrates clear improvements in efficiency and accuracy. Starting from the baseline mAP of 88.4%, FLOPs of 6.7G, and parameters of 2.6M, adding C3k2FC alone raises mAP to 90.1%, while reducing FLOPs to 6.1G and parameters to 2.3M. This enhancement arises from C3k2FC’s partial channel convolutions and gating mechanisms, which alleviate redundancy in parallel branches and bolster local feature capture, proving especially useful for differentiating BB lesions under leaf occlusions and lighting variations. When combined with other modules, such as C3k2FC + SPPF_LSKA (mAP 92.6%, FLOPs 6.4G, parameters 2.6M), C3k2FC + SPPF_LSKA + SlimNeck (mAP 93.1%, FLOPs 6.2G, parameters 2.6M), and the full model C3k2FC + SPPF_LSKA + SlimNeck + LiteHead (mAP 94.1%, FLOPs 5.9G, parameters 2.5M), the module contributes to consistent gains in mAP while maintaining or reducing computational costs, underscoring its role in foundational redundancy mitigation.

The SPPF_LSKA module contributes to refined multi-scale handling and noise suppression. When added to the baseline with SlimNeck (baseline + SPPF_LSKA + SlimNeck: mAP 93.9%, FLOPs 6.9G, parameters 2.9M) or with SlimNeck + LiteHead (baseline + SPPF_LSKA + SlimNeck + LiteHead: mAP 94.4%, FLOPs 6.5G, parameters 2.9M), it elevates mAP through LSKA attention’s refinement of pooling operations, effectively amplifying fine-grained details like striped BB patterns in high-altitude UAV images while managing a modest increase in FLOPs. In combinations involving C3k2FC, such as C3k2FC + SPPF_LSKA (mAP 92.6%, FLOPs 6.4G, parameters 2.6M) and C3k2FC + SPPF_LSKA + SlimNeck (mAP 93.1%, FLOPs 6.2G, parameters 2.6M), SPPF_LSKA further boosts performance by enhancing adaptability to varying scales and background noise, leading to better suppression of irrelevant features in challenging rice field environments.

SlimNeck’s incorporation focuses on optimizing the Neck for computational efficiency. Standalone addition to the baseline yields mAP of 91.7%, FLOPs 6.3G, and parameters 2.6M, with GSConv’s channel mixing and VoVGSCSP’s reuse mechanisms addressing overhead in resource-constrained UAV systems. Combinations like baseline + SlimNeck + LiteHead (mAP 91.9%, FLOPs 6.0G, parameters 2.4M) and baseline + SPPF_LSKA + SlimNeck (mAP 93.9%, FLOPs 6.9G, parameters 2.9M) show reduced FLOPs or improved multi-resolution fusion, ensuring global context extraction without excessive computational demands.

Finally, LiteHead refines detection for rotational and small targets. When added alone to the baseline, it achieves mAP of 92.8%, FLOPs 6.4G, and parameters 2.4M, with LiteHead’s separated BN and dynamic anchors enhancing stability and adaptability to rotated BB lesions amid resolution fluctuations. In various combinations, such as baseline + SlimNeck + LiteHead (mAP 91.9%, FLOPs 6.0G, parameters 2.4M), baseline + SPPF_LSKA + SlimNeck + LiteHead (mAP 94.4%, FLOPs 6.5G, parameters 2.9M), and the complete model (mAP 94.1%, FLOPs 5.9G, parameters 2.5M), LiteHead consistently reduces false detections and supports robust performance.

Overall, the ablation demonstrates that each module contributes incrementally to performance, with synergistic effects from combinations aligning with design goals for lightweight, robust BB detection.

#### 3.2.2 Comparative experiments of different models

To evaluate our improved model for BB detection in UAV-captured rice field images, we compared it against state-of-the-art oriented bounding box (OBB) detection models, including Oriented RCNN, Rotated-Faster-RCNN, S²ANet, RetinaNet-OBB, ReDet, RoI Transformer, YOLOv8-OBB, YOLOv11-OBB, and YOLOv12-OBB. All models were tested on the same dataset under identical hardware conditions. Performance metrics include mean Average Precision (mAP), floating-point operations (FLOPs in G), and parameters (in M). Results are summarized in **Table 3**.

**Table 3:** Comparative results of different OBB detection models.

Models	mAP/%	FLOPS/G	Params/M
Oriented RCNN	88.6	121.6	41.1
Rotated-Faster-RCNN	88.3	91.0	41.1
S²ANet	87.9	77.0	38.6
RetinaNet-OBB	88.0	83.3	36.3
ReDet	88.2	48.3	31.6
RoI-Transformer	88.5	105.0	55.1
YOLOv8-OBB	85.5	7.1	2.8
YOLOv11-OBB	88.4	6.7	2.6
YOLOv12-OBB	87.0	6.2	2.5
Our	94.1	5.9	2.5

Oriented RCNN achieves an mAP of 88.6% with 121.6G FLOPs and 41.1M parameters, leveraging a two-stage detection framework but incurring high computational costs due to its complex region proposal network. Rotated-Faster-RCNN, with an mAP of 88.3%, 91.0G FLOPs, and 41.1M parameters, offers comparable accuracy with reduced computational demand. S²ANet, an anchor-free model, records an mAP of 87.9%, 77.0G FLOPs, and 38.6M parameters, balancing efficiency and accuracy but struggling with small objects in dense rice fields. RetinaNet-OBB, a one-stage model using focal loss, achieves an mAP of 88.0% with 83.3G FLOPs and 36.3M parameters, limited by feature extraction for rotated objects. ReDet, with an mAP of 88.2%, 48.3G FLOPs, and 31.6M parameters, optimizes rotation-invariant features but shows slightly lower accuracy in complex scenarios. RoI Transformer, with an mAP of 88.5%, 105.0G FLOPs, and 55.1M parameters, excels in handling rotated objects via a transformer-based approach but is resource-intensive. Among YOLO-based models, YOLOv8-OBB, with an mAP of 85.5%, 7.1G FLOPs, and 2.8M parameters, provides a lightweight baseline but lags in accuracy due to less advanced feature fusion. YOLOv11-OBB improves to an mAP of 88.4%, 6.7G FLOPs, and 2.6M parameters with better gradient flow and multi-scale handling. YOLOv12-OBB, with an mAP of 87.0%, 6.2G FLOPs, and 2.5M parameters, gains efficiency but falls short in accuracy for fine-grained detection. Our model achieves the highest mAP of 94.1% with the lowest FLOPs (5.9G) and parameters (2.5M), outperforming all comparators. This superiority stems from optimizations like C3k2FC for redundancy reduction, SPPF_LSKA for noise suppression, SlimNeck for efficient fusion, and LiteHead for rotational adaptation, ensuring robust performance in challenging rice field scenarios with minimal resource demands.

#### 3.2.3 Results of pruning experiments

To evaluate the impact of the Layer-adaptive Magnitude-based Pruning (LAMP) method on the improved model, we conducted pruning experiments at varying rates (1.5, 2.0, 2.5, and 3.0), where the pruning rate represents the adaptive magnitude threshold factor applied globally to weights, with higher values indicating more aggressive pruning. The improved model (unpruned) serves as the reference, and all evaluations were performed under the same dataset and environment conditions. Metrics include mean Average Precision (mAP), FLOPs (G), and Parameters (M). The results are summarized in **Table 4**.

**Table 4:** Pruning experiment results at varying pruning rates.

Pruning Rate	mAP	FLOPS/G	Params/M
Base	94.1%	5.9	2.5
1.5	93.2%	4.2	1.8
2.0	93.1%	3.5	1.4
2.5	80.5%	2.8	1.1
3.0	59.7%	2.3	0.9

The baseline achieves mAP of 94.1% with FLOPs of 5.9G and parameters of 2.5M. At a pruning rate of 1.5, mAP slightly drops to 93.2%, but FLOPs reduce to 4.2G and parameters to 1.8M, demonstrating minimal accuracy loss with substantial efficiency gains due to LAMP’s layer-adaptive scoring, which preserves critical connections in early layers for fine-grained BB lesion capture. Increasing to 2.0 yields mAP of 93.1%, FLOPs 3.5G, and parameters 1.4M, maintaining robust performance amid lighting variations and occlusions by dynamically adjusting sparsity to protect feature extraction pathways.

However, at 2.5, mAP declines more noticeably to 80.5%, with FLOPs 2.8G and parameters 1.1M, indicating a threshold where aggressive pruning begins to erode semantic representation. At 3.0, mAP plummets to 59.7%, FLOPs 2.3G, and parameters 0.9M, highlighting severe degradation from overpruning, which amplifies false negatives in noisy rice field environments. These findings underscore LAMP’s effectiveness at a pruning rate of 2.0 for balancing compression and accuracy, ideal for resource-constrained UAV deployments, while higher rates risk compromising BB detection reliability. The final model uses LAMP pruning at rate 2.0 for optimal balance.

#### 3.2.4 Detection performance in different scenarios

**Figure 7:**
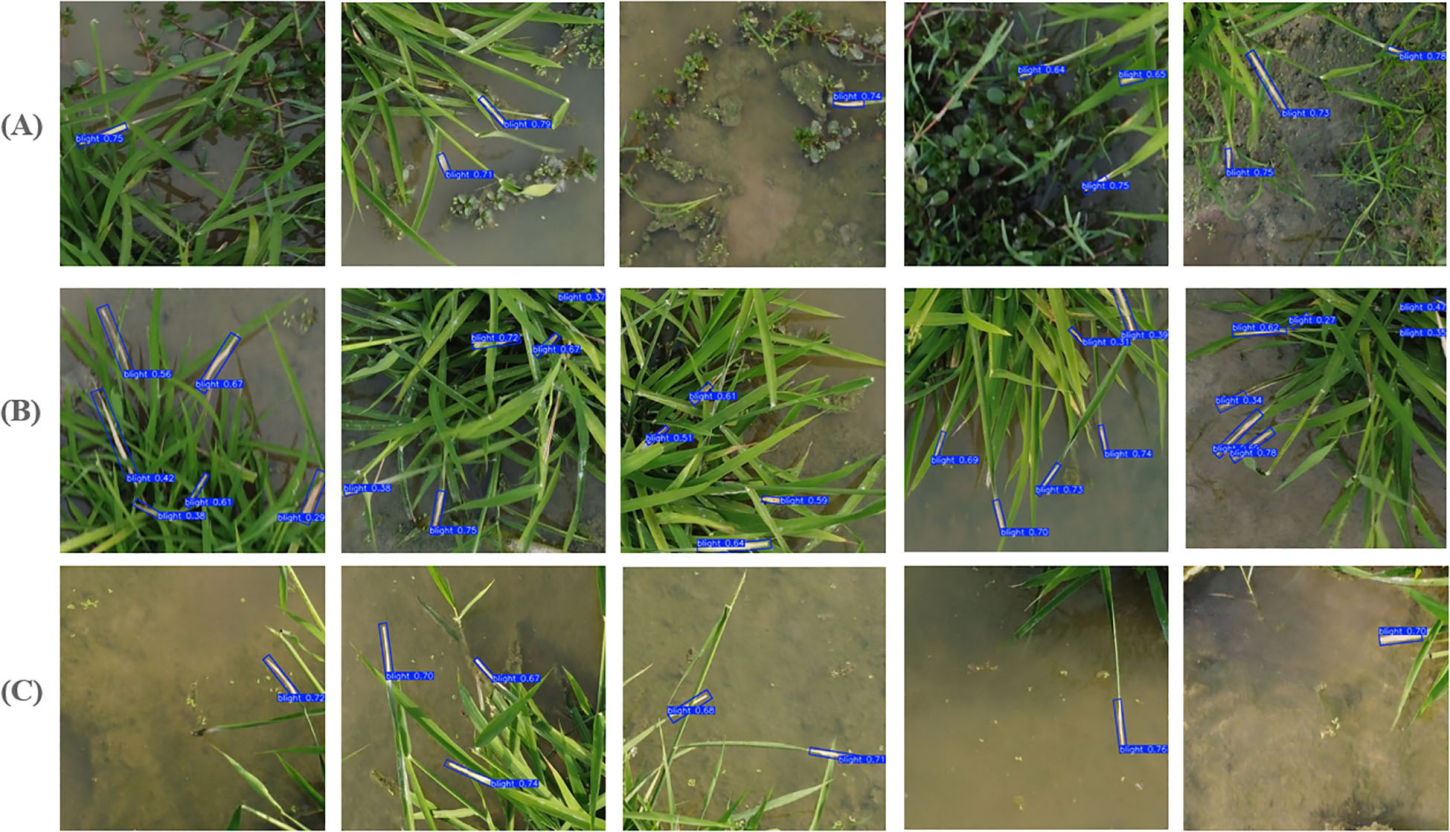
Performance comparison of recent YOLO-based OBB detection models across different scenarios. **(A)** Detection under complex backgrounds. **(B)** Detection under dense disease conditions. **(C)** Detection under strong midday lighting.

To further evaluate the performance of the proposed BB detection model in different scenarios, we selected 50 images from the preaugmentation test set for each of the following conditions: detection under complex backgrounds, dense disease conditions, and strong midday lighting. The results, along with comparisons to recent YOLO-based OBB detection models (YOLOv8-OBB, YOLOv11-OBB, and YOLOv12-OBB), are summarized in **Table 5**. **Figure 7** visualizes the performance comparisons across the various models and conditions. The red circle marks locations of false detections, while the red square highlights missed detections.

**Table 5:** Comparison of recent YOLO-Based OBB detection models across different scenarios.

Models	Complex backgrounds	Dense disease	Strong midday lighting
YOLOv8-OBB	79.7%	81.5%	87.5%
YOLOv11-OBB	80.6%	83.7%	88.4%
YOLOv12-OBB	82.1%	87.2%	86.3%
Our	93.7%	94.5%	93.8%

**Figure 8:**
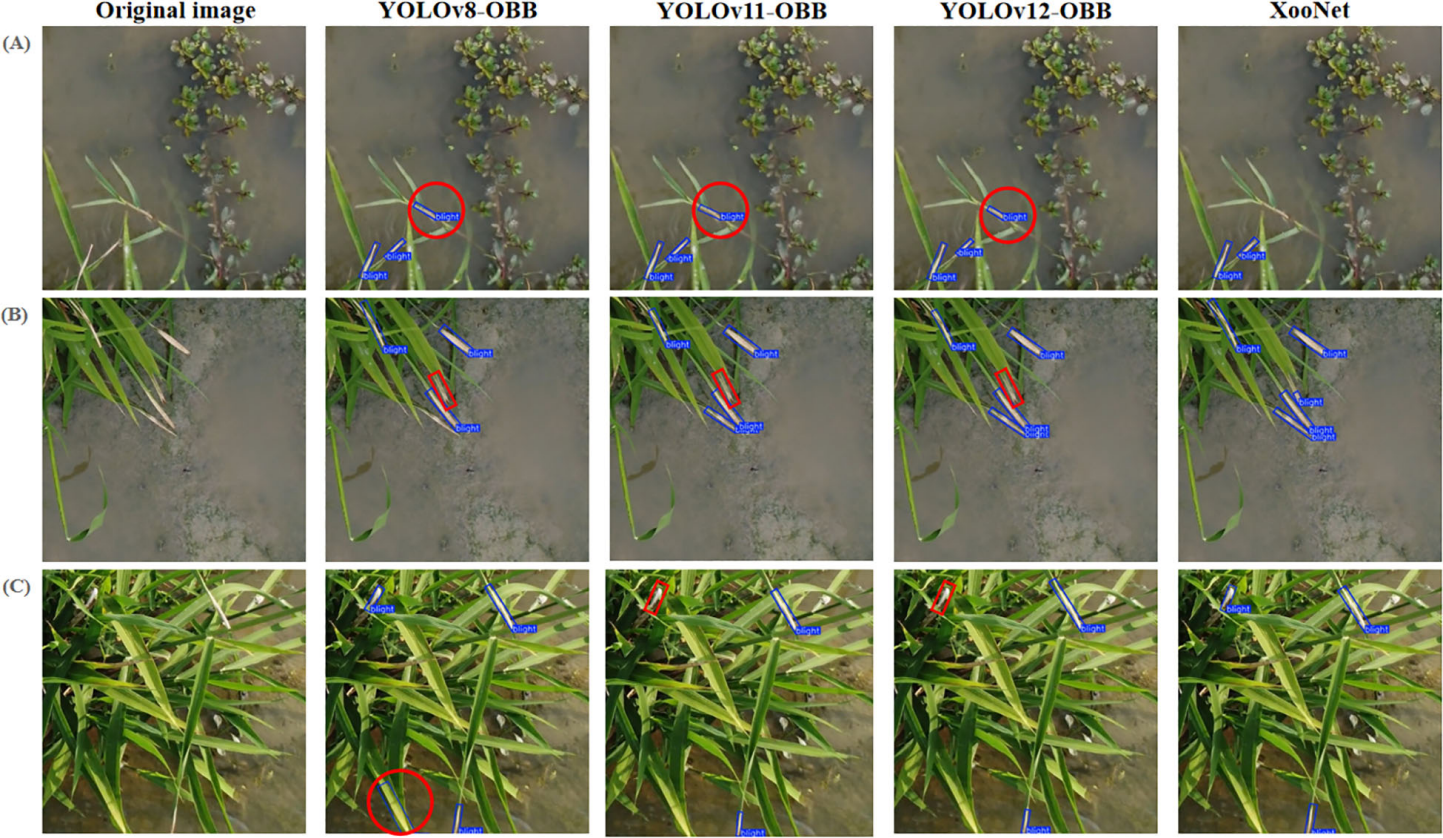
Detection results in different scenarios. **(A)** Detection under complex backgrounds. **(B)** Detection under dense disease conditions. **(C)** Detection under strong midday lighting.

Experimental results demonstrate that the proposed model outperforms the other YOLO-based models in all scenarios. As shown in **Table 5**, our model achieves the highest detection performance across complex backgrounds (93.7%), dense disease conditions (94.5%), and strong midday lighting (93.8%), outperforming YOLOv8-OBB, YOLOv11-OBB, and YOLOv12-OBB. These results highlight the robustness of our model in handling diverse and challenging environmental conditions. We randomly selected detection results from a variety of environmental conditions, as shown in **Figure 8**.

As shown in **Figure 8**, the proposed model effectively addresses these challenges, demonstrating robust performance in complex backgrounds, dense disease scenarios, and varying lighting conditions, underscoring its potential for reliable and accurate disease detection.

#### 3.2.5 Detection performance on edge computing devices

To evaluate the performance of the model deployed on edge computing devices, we tested a 60-second video captured by a UAV of wild rice infected with BB. The video had a resolution of 1920x1080 pixels. The improved model achieved a detection speed of 7.5 fps in the unaccelerated mode, and 21.3 fps with TensorRT acceleration, resulting in an improvement of 13.8 fps. This represents a 2.84-fold increase in performance. In contrast, the YOLOv11-OBB model showed an improvement of 9.3 fps, with a 2.69-fold increase in performance after applying TensorRT acceleration. Prior to acceleration, the detection speed of the improved model was constrained by the limited computational power of the Jetson Nano device. However, after applying TensorRT acceleration, the detection speed of the improved model reached 21.3 fps, achieving a significant increase in performance. These results demonstrate that the improved model, with TensorRT acceleration, significantly enhances detection speed, making it well-suited to meet the performance requirements for deployment on edge computing devices.

### 3.3 Assessment of BB screening performance

#### 3.3.1 Analysis of BB screening accuracy

The experimental results show that the method accurately assessed 117 varieties, with 3 plants misclassified, resulting in an overall accuracy of 97.5%. This performance is sufficient for disease resistance breeding applications. Further analysis of disease counting performance revealed that the method correctly counted the disease in 112 plants, with 8 varieties showing varying degrees of miscounts, yielding an overall accuracy of 93.3%. To better understand the source of these errors, the miscounted samples were analyzed.

From **Table 6**, it is evident that five samples (IDs 025, 034, 045, 064, 089) were missed detection due to leaf disturbance. The cause of this was the vibrations and airflow from the UAV’s propellers, which caused the leaves to move vigorously. This disturbance resulted in blurred and overlapping leaves in the images, making it difficult for the detection model to accurately detect and locate the disease. Additionally, some wild rice leaves exceeded 50 cm in length, which made them more susceptible to wind, further increasing the chances of missed detection. Three samples (IDs 094, 096, 109) were misclassified due to the presence of dead leaves that were not cleared in time. The visual similarity between dead leaves and BB led to false positives during the disease detection process.

**Table 6:** Disease detection miscount analysis and causes.

Sample ID	Actual disease count	Disease count by this method	Cause of miscount
025	9	8	Missed detection due to leaf disturbance
034	4	2	Missed detection due to leaf disturbance
045	2	0	Missed detection due to leaf disturbance
064	6	4	Missed detection due to leaf disturbance
089	8	7	Missed detection due to leaf disturbance
094	3	5	Misclassification due to dead leaves not cleared in time
096	7	6	Misclassification due to dead leaves not cleared in time
109	7	9	Misclassification due to dead leaves not cleared in time

#### 3.3.2 Analysis of practical application test results

The experimental results showed that the method correctly screened 47 wild rice varieties, with 3 varieties misclassified, resulting in an overall accuracy of 94.0%. This performance meets the requirements for disease resistance breeding applications. Further analysis of the disease counting performance revealed that the method accurately counted the disease in 43 plants, with 7 plants showing varying degrees of miscounts, yielding an overall accuracy of 86.0%. To better understand the source of these errors, the miscounted samples were analyzed.

From **Table 7**, it is clear that four samples (IDs 005, 009, 017, 041) were missed detection due to occlusion. This was primarily caused by the failure to remove healthy leaves, which grew too quickly and obscured the diseased targets. As the UAV captured images from above, these occluded targets could not be effectively detected, making it difficult for the detection model to identify them. Additionally, three samples (IDs 020, 024) were missed detection due to leaf disturbance. One sample (ID 043) was misclassified due to the presence of dead leaves that were not cleared in the field.

**Table 7:** Miscount analysis and causes in field application test.

Sample ID	Actual disease count	Disease count by this method	Cause of miscount
005	4	1	Occlusion
009	5	2	Occlusion
017	6	3	Occlusion
020	6	5	Missed detection due to leaf disturbance
024	8	7	Missed detection due to leaf disturbance
041	8	4	Occlusion
043	3	5	Misclassification due to dead leaves not cleared

### 3.4 Summary of experimental results

In summary, the comprehensive evaluation results from **Tables 2** through 5 demonstrate that XooNet achieves a superior balance between detection accuracy and computational efficiency. Compared to the baseline YOLOv11-OBB, the final optimized model improved the mAP by 4.7% (increasing from 88.4% to 93.1%) while significantly reducing model complexity. Furthermore, field deployment tests confirmed that with TensorRT acceleration on the Nvidia Jetson Nano, the model achieves an inference speed of 21.3 FPS. These findings validate that XooNet meets the strict requirements for real-time, high-precision bacterial blight screening in wild rice fields.

## 4 Discussion

Although significant progress has been made in the detection of rice BB, existing methods often fail to adequately consider the critical issue of equipment costs. Some techniques, while offering high detection accuracy, rely on expensive equipment and require specialized operators, limiting their practical application. Additionally, some methods are computationally complex, which restricts their use in routine breeding practices. In contrast, the XooNet method presented in this study employs low-cost UAVs and lightweight deep learning models, maintaining high detection precision while reducing equipment and operational costs, making it more suitable for practical breeding applications.

One key advantage of this method is that it offers a cost-effective solution. Unlike other methods that rely on expensive hyperspectral equipment or specialized hardware, XooNet utilizes commercially available UAVs, making it more affordable and accessible for breeding laboratories with limited budgets. For example, hyperspectral imaging systems that cost over $80,000 are often used in some studies for this task, but such systems are not feasible for routine field use due to their high cost and technical requirements. In contrast, XooNet provides an equally effective solution at a fraction of the cost, enabling broader adoption in real-world breeding scenarios.

Another key advantage is its robust performance in complex field environments. One of the standout features of XooNet is its lightweight OBB detection algorithm, which has been specifically designed for UAV-based BB disease screening. This algorithm ensures stable performance in the dynamic and often challenging conditions found in real-world rice fields. It can handle detection under complex backgrounds, such as weeds, dense disease conditions, and strong midday lighting. This field performance ensures that XooNet remains reliable in diverse environmental conditions, making it ideal for BB-resistance breeding.

Despite the promising results, certain considerations should be observed during the application of this method. First, strict adherence to inoculation protocols and field management requirements is essential. According to the Rice Disease Resistance Evaluation Technical Standards, it is necessary to manage the disease plots carefully to avoid external factors influencing the experimental results. Although the accuracy of the proposed method is high, errors were observed primarily due to improper inoculation procedures (e.g., using leaves that were too long or too short) or poor field management (e.g., failure to clear uninfected leaves or dead leaves promptly). Therefore, improving field management practices is crucial to enhance reliability. Second, the quality of the UAVs used for image capture plays a significant role. The DJI Mini 2 UAV used in this study supports only 2x zoom, necessitating a lower flying altitude. However, subsequent tests with the DJI Mini 4 Pro UAV showed that increasing the zoom and flight altitude significantly reduced leaf disturbance, thereby improving screening accuracy.

While XooNet has demonstrated encouraging results in accuracy and efficiency, it is important to acknowledge a limitation regarding the data distribution. The dataset utilized in this study was constructed by pooling and randomly splitting images collected over two growing seasons (2023 and 2024). Strict independent cross-year validation (i.e., training on one year and testing on the other) was not performed in this study. Consequently, while the model demonstrated high accuracy within the tested data distribution, its generalizability to completely unseen years or varying ecological regions with significantly different environmental characteristics requires further verification.

To address these limitations and enhance practical applicability and robustness in field conditions, future research will focus on several key directions. First, we plan to enhance model performance through data diversity and reinforcement learning. We aim to expand the diversity of the data covering various dimensions such as different wild rice varieties, fungal strains, experimental environments, and growth stages. Additionally, introducing deep reinforcement learning methods will help address errors caused by dynamic factors such as leaf vibrations and airflow disturbances. Second, future work could integrate the current method with genotypic data to advance genomic selection (GS) techniques in breeding. Combining phenotypic data with DNA markers can help accurately select high-quality germplasm with disease-resistant genes. Finally, we aim to integrate environmental factors, such as climate data and soil conditions, with disease resistance phenotypic data. This approach will help develop predictive models to assess how wild rice varieties perform under different ecological conditions, optimizing breeding strategies and adapting to climate change.

Despite certain limitations, the XooNet method offers valuable technical guidance for automated, precise disease screening in disease-resistant breeding applications. The method has already contributed to several wild rice BB resistance breeding projects, providing an efficient screening tool. As the technology continues to improve and gain wider adoption, XooNet will further demonstrate its reliability and effectiveness, providing strong technical support for rice disease resistance breeding.

## 5 Conclusions

This study introduces XooNet, a novel UAV-based method for automated BB resistance screening in wild rice, which classifies wild rice into several levels based on BB resistance. To enable this method, a high-precision and lightweight OBB detection algorithm for BB in wild rice is developed. Experimental results show that the screening method achieved an accuracy of 97.5%. Through the implementation of the lightweight OBB algorithm and LAMP pruning, the final detection model achieved an accuracy of 93.1% with a parameter size of 1.4M and a computational complexity of 3.5 GFLOPs. The XooNet method offers a cost-effective and efficient solution for large-scale BB resistance screening, overcoming the limitations of traditional methods and existing UAV-based approaches. It provides strong support for the integration of wild rice resistance genes into breeding programs, enabling rapid identification of resistant varieties. Future research will aim to integrate phenotypic and genotypic data, along with environmental factors, to enhance the accuracy and applicability of this method.

## Data Availability

The raw data supporting the conclusions of this article will be made available by the authors, without undue reservation.
